# 737 Burn Data Management and Usage Across Canada: A Nationwide Survey

**DOI:** 10.1093/jbcr/irad045.212

**Published:** 2023-05-15

**Authors:** Eduardo Gus, Thrmiga Sathiyamoorthy, Jane Zhu, Joel Fish

**Affiliations:** The Hospital for Sick Children, Toronto, Ontario; University of Toronto, Toronto, Ontario; University of Toronto, Toronto, Ontario; The Hospital for Sick Children, Toronto, Ontario; The Hospital for Sick Children, Toronto, Ontario; University of Toronto, Toronto, Ontario; University of Toronto, Toronto, Ontario; The Hospital for Sick Children, Toronto, Ontario; The Hospital for Sick Children, Toronto, Ontario; University of Toronto, Toronto, Ontario; University of Toronto, Toronto, Ontario; The Hospital for Sick Children, Toronto, Ontario; The Hospital for Sick Children, Toronto, Ontario; University of Toronto, Toronto, Ontario; University of Toronto, Toronto, Ontario; The Hospital for Sick Children, Toronto, Ontario

## Abstract

**Introduction:**

Burn injuries account for significant morbidity in the Canadian population. Registries are an invaluable resource for the collection and interpretation of data surrounding disease and injury, answering research questions, and engendering benchmarking of quality-of-care indicators. Despite the rapid growth of registry science in the past decade in other jurisdictions, there has yet to be a burn registry developed for Canadian burn centres.

The objectives for this project are to characterize the current state of burn data collection, analysis, and dissemination, and to explore the interest of various burn centres in contributing to a Canada-wide burn registry.

**Methods:**

A 23-item mixed methods survey was created using REDCap and electronically delivered to burn directors or research managers of 22 burn centres across Canada. Descriptive statistics and thematic analysis were used for quantitative and qualitative analysis, respectively.

**Results:**

Sixteen (72%) complete survey responses were received. Many centres treat both pediatric and adult patients, and all centres collect acute inpatient burn data. Types of data collected included epidemiology (88%), outcomes (69%) and quality of care indicators (44%). Most burn units (56%) collected data using their hospital’s database. Data was largely used for institutional learning purposes (81%). Routine use included quality improvement (69%), clinical research (50%), and patient care (50%). A minority of institutions report using their data for external purposes, such as conference presentations (31%) or journal publications (19%). While all units are currently collecting data, half of the institutions did not analyze their data, and a majority (73%) of institutions did not benchmark their data against other institutions.

Feedback for creating a registry included overall strong support for the initiative. The most significant barrier perceived towards implementing a registry was cost, time, and human resources. Suggested measures to facilitate registry development included standardizing data entry and engagement with national and provincial institutions.

**Conclusions:**

Although all Canadian burn centres are currently collecting data for institutional and quality improvement purposes, it is not routinely shared or used for benchmarking purposes. Burn centres demonstrated interest and support in contributing to a novel Canadian burn registry.

**Applicability of Research to Practice:**

Future research may expand on initial themes outlined in our study to provide groundwork for the formation of a novel Canadian burn registry.

